# Psychometric Validation of the Revised Family Affluence Scale: a Latent Variable Approach

**DOI:** 10.1007/s12187-015-9339-x

**Published:** 2015-10-18

**Authors:** Torbjørn Torsheim, Franco Cavallo, Kate Ann Levin, Christina Schnohr, Joanna Mazur, Birgit Niclasen, Candace Currie, the FAS Development Study Group

**Affiliations:** Department of Psychosocial Science, University of Bergen, Postbox 7800, 5020 Bergen, Norway; Department of Public Health and Pediatrics, University of Turin, Turin, Italy; NHS Greater Glasgow & Clyde, Public Health Directorate, Glasgow, G12 0HX UK; Department of Public Health, University of Copenhagen, Copenhagen, Denmark; Department of Child and Adolescent Health, Institute of Mother and Child, Warsaw, Poland; National Institute of Public Health, University of Southern Denmark, Odense, Denmark; School of Medicine, Medical & Biological Sciences, University of St. Andrews, Scotland, UK

**Keywords:** SES, FASIII, Measurement, Adolescence, HBSC

## Abstract

The aim was to develop and test a brief revised version of the family affluence scale. A total of 7120 students from Denmark, Greenland, Italy, Norway, Poland, Romania, Scotland and Slovakia reported on a list of 16 potential indicators of affluence. Responses were subject to item screening and test of dimensionality. Bifactor analysis revealed a strong general factor of affluence in all countries, but with additional specific factors in all countries. The specific factors mainly reflected overlapping item content. Item screening was conducted to eliminate items with low discrimination and local dependence, reducing the number of items from sixteen to six: Number of computers, number of cars, own bedroom, holidays abroad, dishwasher, and bathroom. The six-item version was estimated with Samejima’s graded response model, and tested for differential item functioning by country. Three of the six items were invariant across countries, thus anchoring the scale to a common metric across countries. The six-item scale correlated with parental reported income groups in six out of eight countries. Findings support a revision to six items in the family affluence scale.

## Introduction

Research on health inequality, child poverty and living standards rely on quantitative indicators of family wealth. Household income or consumption expenditure is in some studies the preferred indicator of wealth, but additional indicators are needed in studies that involve child or adolescent self-report. Child and adolescent surveys typically do not include parents as informants, and for children and adolescents, accurate information about parental income might not be accessible. An alternative to income-based measures of family wealth, sometimes referred to as the “assets approach” (Howe et al. 2008; Sahn and Stifel 2003), is to ask the child or their parents directly about the material conditions in the family (Doku et al. 2010; Traynor and Raykov 2013; Wardle et al. 2002). The assets approach requires children and adolescents to report on family ownership of goods and/or families access to services that are required for an acceptable standard of living. An index of family wealth is obtained by summing across indicators (Doku et al. 2010; Wardle et al. 2002). In general, assets-based indices can be used to assign a measure or ‘score’ of wealth along a gradient, or to create a cutoff that classifies families as below or above the poverty line, either defined by statistical or by consensual principles (Mack and Lansley 1985). Indices based on the “assets approach” are flexible in that they can be used as the outcome of interest or as a stratification variable in studies of health inequality.

The current study presents findings on the development and revision of the family affluence scale (FAS), a brief assets-based measure of family wealth that was designed for use in adolescent surveys. The family affluence scale (Currie et al. 1997, 2008) was developed within the “Health Behaviour in School-Aged Children study” (HBSC) as measure of family wealth. FAS was initially devised for use at a national level (Currie et al. 1997) but soon after adopted for the cross-national HBSC, as a valid comparative measure of family affluence across Europe and North America. Since the initial development, FAS has been widely used in research papers (Currie et al. 2008), indicating its relevance within the adolescent research community. Studies have documented the measurement properties of the scale among school-aged children and adolescents in geographically diverse samples (Boudreau and Poulin 2009; Currie et al. 1997; Kehoe and O’Hare 2010; Liu et al. 2012; Molcho et al. 2007).

Like other asset-based indicators, the family affluence scale needs to be revised in order to reflect the changing societal patterns of consumption and lifestyles of families with adolescents. To illustrate, the first version of the family affluence scale (FAS I) (Currie et al. 1997) included three items: number of cars, number of vacations and having own bedroom. In 2002, to increase validity, FAS II also included computer ownership. Current economic and technological developments necessitate further development of the family affluence scale. The need for scale revision is spurred by indications that the value of existing indicators in the FAS II has changed over time. For example, computers once a luxury item, is now owned universally by almost all households within Europe and North America. In line with this, Schnohr et al. showed significant parameter drift for the computer item from 2002 to 2010 (Schnohr et al. 2013), probably reflecting that not only the *number* of computers in the family, but also the *value* of computers have changed over time. The changing value of material goods highlights the difficulty in comparing inequalities over time when the distribution, composition and possibly the validity of the overall FAS score changes over time.

In addition to changes in the meaning of FAS over time, there is also the issue of differences between countries and the need for culturally sensitive indicators. Studies have pointed to cross-national differences in the psychometric properties of the indicators included in FAS I (Batista-Foguet et al. 2004), and FAS II (Schnohr et al. 2008, 2013). Schnohr and colleagues (Schnohr et al. 2008) reported a conditional effect of country, suggesting that a subset of items in the scale worked differently across countries, leading to potential bias in comparisons across countries. Some of these differences can be accounted for through common item equating (Makransky et al. 2014), but such equating requires at least two anchor items to establish a common metric. Schnohr and colleagues (2008) did not identify any such anchor items.

Development and revision of asset-based indices can be guided by multiple criteria. Within the social policy field, asset-based indicators have been developed from a consensual perspective (Mack and Lansley 1985). In this line of studies, indicators are selected on the basis of what the majority reports as necessities to obtain an acceptable minimum living standard, and the selected indicators are then used for establishing poverty lines. Measures based on the consensual approach maximize information at the lower end of the wealth distribution, which is the relevant part of the distribution for studies of child poverty. For measures that aim to cover the entire dimension, from low to high affluence, the consensual minimum standard approach might be less appropriate, as the method would tend to select indicators of low affluence, but not indicators of high affluence. In a situation where the entire dimension of affluence is at interest, for example in studies of health inequality and living standards, latent variable models (Knott and Bartholomew 1999) provide additional criteria for scale development and revision (Traynor and Raykov 2013; Vandemoortele 2013). The simple argument in a latent variable model of family affluence is that family affluence *makes* families buy more cars, computers, and holidays. It follows from this argument that cars, computers, and holidays are observed manifestations of family affluence. As implied by several authors, a latent variable also implies *causation* (Bollen and Bauldry 2011; Borsboom et al. 2003). If affluence is a cause of purchasing power, an increase in affluence also increases the probability of having more cars, more vacations, and more home space.

Since family affluence is seen as a *cause* of consumption across goods and services, one would expect to observe common variance across such indicators for a given family. As a statistical criterion, indicators would be selected based on the degree of covariance between economic goods and services, the *internal consistency* criterion (Cronbach 1951). Applying this criterion, items that correlate weakly with other goods and services might be poor indicators of affluence. Goods and services that do not covary with the presence of other goods and services in the family are, according to this perspective, not indicators of the family affluence.

Following the psychometric principles of construct validation (e.g. (Cronbach and Meehl 1955; Nunnally and Bernstein 1994)), a valid measure of family affluence should also relate systematically to other constructs, such as household income. All else being equal, it would be reasonable to expect a strong covariance between adolescents’ reports of affluence and corresponding parental reports of household income.

### The Current Study

While the published literature generally indicates a high concurrent validity in terms of relationship with other outcomes (Currie et al. 2008), the construct validity of FAS requires further attention. To develop FAS along a reflective measurement model, an important object is to increase internal consistency of the scale, which in the existing four-item version of FAS (FAS II) is low (Schnohr et al. 2008). One way of increasing the reliability of the scale is to increase the number of items and to remove items with low information. Importantly, new items should reflect affluence, and should be statistically and theoretically associated with the existing indicators of the scale. With reference to the problem of comparability across countries there is a particular need for anchor items that can be used to anchor each country’s score to a common metric.

The current study presents initial findings from a validation study aimed to revise and develop a new and valid Family Affluence Scale (FAS III), comprising items that reflect current economic trends, technological advances, as well as cultural, social and geographical norms in consumption across Europe and North America. The objective of the current study was to develop a brief six-item scale for use in cross-national comparative surveys of school-aged children. The six-item limit was based on the premise that the scale should be feasible for inclusion in large cross-national surveys of school-aged children and adolescents where the broad coverage often imposes strong limits on the number of indicators.

## Methods

### Procedure

A protocol was produced including a self-complete questionnaire survey and guidelines on sampling and administration of the survey within schools. Data were collected in Denmark, Italy, Norway, Poland, Romania, Scotland, Greenland and Slovakia. According to the protocol, the target student sample size for was 500 students, covering age groups 11, 13 and 15 in each country. In addition, 200 of the 500 children were surveyed 4 weeks later for reliability analysis. The desired sample size of 500 was selected to represent a lower bound for obtaining stable estimates in item response theory models (IRT) and modern limited information methods such as robust weighted least squares (WLSMV) (Flora and Curran 2004). A protocol was also produced for a parental questionnaire. The purpose was to cross-validate the child’s response to each FAS item with their parent and also to validate FAS against other measures of the family SES, including family income. Researchers worked with a school class (or sometimes more than one class at a time) of pupils (11-, 13- or 15- year olds). In each of the participating countries study protocol were reviewed and accepted through relevant ethical bodies.

### Sample

As shown in Table 1, the student sample consisted of 3867 students from Denmark (*M*_age_ = 13.73), 517 students from Italy (*M*_age_ = 13.87), 390 students from Norway (*M*_age_ = 13.07), 493 students from Poland (*M*_age_ = 13.03), 172 students from Romania (*M*_age_ = 13.96), 564 students from Scotland (*M*_age_ = 12.97), 613 students from Slovakia (*M*_age_ = 12.93), and 504 students from Greenland (*M*_age_ = 13.45). The Danish student sample size was bigger than in the other countries, as the Danish survey was part of a nationwide survey on health inequality. Parents of 2013 students across all countries participated in a separate survey, while 994 students participated in the retest survey.

**Table 1:** Sample size by country and survey

	Student survey	Retest survey	Parent survey
Denmark	3867	_	333
Italy	517	164	463
Norway	390	219	267
Poland	493	172	305
Romania	172	_	104
Scotland	564	234	206
Slovakia	613	114	270
Greenland	504	91	65
Total	7120	994	2013

### Measurement

The Appendix displays the 16 items that were tested in the current study. Principle Investigators in 41 countries were asked to propose new candidate items to be included in FAS III, based on their knowledge and expertise about their country and the scientific area. In all, 19 of the 43 HBSC countries responded with proposed new items. Countries spanned Europe and included Eastern, Northern, Western and Southern European countries as well as Canada and the US. A scan of the literature was also carried out and additional items added to the list.

### Analysis

The objective of the data analysis was to develop a revised unidimensional family affluence scale, based on a reflective measurement model, where included items reflect the underlying wealth of the family. By default, we expected all indicators to be reflections of family affluence. However, as some items cover similar or overlapping content (for example washer-dryer, holiday- holidays abroad), specific sub-factors were also expected.

We analysed data in an item *screening* stage and a scale *testing* stage. The objective of the screening stage was to reduce the number of items from an initial pool of 16 items to a unidimensional subset of about six items. The screening stage removed items that violated assumptions of unidimensionality, and items that showed unacceptably low discrimination power. In line with suggestions in the literature, to identify local dependence and proper discrimination, we used item bifactor analysis (Reise et al. 2007) using the robust weighted least square estimator (WLSMV) for categorical dependent variables. The bifactor rotation (Jennrich and Bentler 2011) extracts a general factor (g-factor) reflected by all indicators, and specific factors that reflect the residual covariance between subsets of indicators. In the context of testing unidimensionality, the distinction between general and specific factors is a valuable property: The g-factor can be seen as the default expectation for a unidimensional construct, whereas any specific factors point to violations of unidimensionality. The results from a bifactor analysis, therefore, can indicate item redundancy and violation of unidimensionality. Other commonly used factor analytic procedures can also provide such information, but since these methods tend to use a rotated factor solution, essential unidimensionality must be inferred indirectly through information about the correlation between the factors.

Based on the subset of items identified through the screening stage, the objective of the scale testing stage was to assess model fit using item response theory models, and to test for differential item functioning (DIF). Country DIF would present if two persons with *identical* family affluence but from different countries, responded differently to the same indicator of affluence. The presence of country DIF implies that the indicator works differently in different countries. The objective of the differential item functioning test was to identify a subset of items that could work as *anchor items* for establishing a comparable metric across countries. To be able to compare scores directly, ideally, all items should be invariant across countries, but full measurement invariance is difficult to achieve in comparative research. We analysed differential item functioning (DIF) across samples, using item response theory (IRT) models to provide initial student estimates of family affluence. Item response theory (IRT) (Lord 1980) is a family of models for measurement of latent variables. The IRT analysis models the probability of categorical item responses as a function of the underlying continuous trait or dimension. In the current study, the probability of responding that your family has at least two cars is a function of the unobserved family affluence level. The higher family affluence, therefore, the higher the probability of reporting having “two cars”, compared to “one car” or “no car”.

The DIF analysis and equating was conducted using the LORDIF R package (Choi et al. 2011). The LORDIF R package uses an iterative procedure to detect items that work differently across groups. The method uses IRT estimated trait-scores, rather than the observed summed score as the conditioning variable in ordinal regression analysis, with items treated as ordinal dependent variables. For items exhibiting DIF with respect to the ‘country’ factor, country will be related to item responses, even after conditioning on estimated trait levels, i.e. people with the same level of trait but from different countries respond differently to an item. Finally, as an external validation, we tested the association between scale scores and parental income groups. The total scale score was obtained through a simple summation of item scores.

## Results

### Item Screening

Table 2 shows the results from the bifactor analysis. The bifactor model suggested a general factor in all samples, but the standardized loadings on the general factor differed across indicators from an average of 0.70 for the “dishwasher” item to 0.32 for “holiday home”. In the Norwegian sample, estimates of factor loadings in the bifactor analysis could not be obtained for “own bedroom”, “own bed”, “washer” and “dishwasher”, as these questions were answered in the same way by almost the entire Norwegian sample. The “Internet” item caused estimation problems in the Romanian and Slovakian sample. Estimation including the “washer” item encountered problems in the Scottish and Slovakian sample.

**Table 2:** Exploratory bifactor analysis with standardized item loadings on general common factor

	Den	Grl	Ita	Nor	Pol	Rom	Sco	Slo	Mean
Dishwasher	0.75	0.86	0.56	–	0.58	0.82	0.68	0.65	0.70
Internet	0.62	0.52	0.59	–	0.55	–	0.86	–	0.63
Bathroom	0.54	0.59	0.71	0.71	0.44	0.71	0.60	0.61	0.61
Cars	0.51	0.62	0.72	0.62	0.53	0.51	0.67	0.57	0.59
Computers	0.66	0.56	0.55	0.48	0.66	0.70	0.39	0.52	0.57
Paid workers	0.31	0.38	0.68	0.44	0.47	0.80	0.46	0.56	0.51
Outdoor area	0.67	NA	0.60	0.73	0.36	0.22	0.54	0.34	0.49
Own bedroom	0.60	0.29	0.43	–	0.46	0.50	0.30	0.41	0.43
Tumble dryer	0.70	0.54	0.29	0.37	0.39	–	0.34	0.36	0.42
Washer	0.84	0.22	0.23	–	0.46	0.35	–	–	0.42
Holiday abroad	0.19	0.34	0.33	0.30	0.53	0.53	0.55	0.48	0.41
Own bed	0.54	0.14	0.58	–	0.37	–	–	0.35	0.40
Holiday	0.27	0.27	0.33	0.35	0.58	0.20	0.66	0.36	0.38
Own computer	0.59	0.36	0.31	0.28	0.56	0.46	0.01	–	0.37
iPod	0.30	0.24	0.31	0.32	0.33	0.59	0.28	0.29	0.33
Holiday home	0.13	0.26	0.23	0.40	0.39	0.48	0.28	0.38	0.32

*NA* item not included.—Item omitted due to numerical inadmissible solution

As expected from the bifactor approach, items with content overlap loaded on specific factors in addition to the main general factor. The number of specific factors varied across samples. In seven out of eight countries “holiday” and “holiday abroad” was one of the specific factors. “Bedroom” and “own bed” emerged as factors in four out of eight samples. The “computer” and “own computer” emerged as a specific factor in four out eight countries. The “dryer”/”washer” items emerged as a specific factor in two countries. Finally, there were also a few country specific factors. The presence of specific factors indicated local dependency between items.

Based on the bifactor analysis items were screened to accomplish a unidimensional model with locally independent items. For the groups of items that loaded on a specific factor one of the items was retained and the other items that loaded on the specific factor was excluded from the scale. This led to keeping “holiday abroad” (removal of “holiday”), “own bedroom” (removing “own bed”), “dishwasher” (removing “washer” and “dryer”), “computer”, (removing “Internet”, “own computer” and “iPod”).

### Scale Testing

**Fig. 1:**
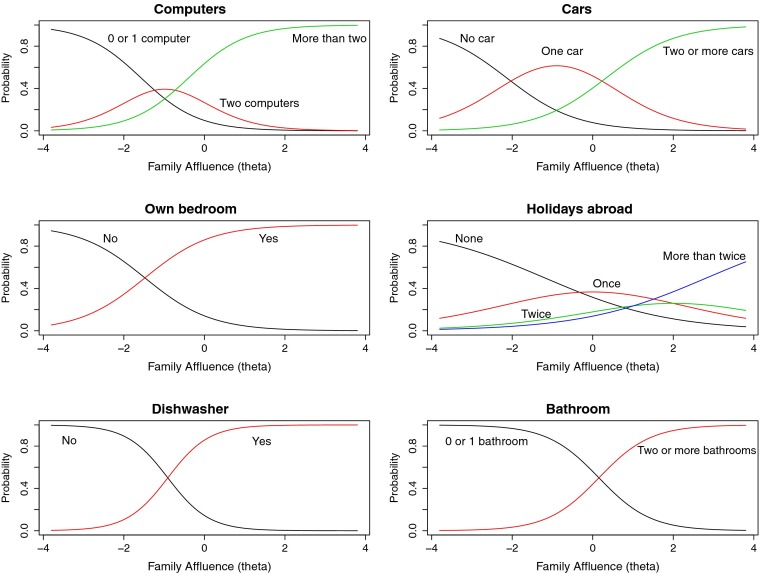
Item characteristic curves for FAS III

From the original pool of 16 assets, six assets were retained as potential items unidimensional scale: Number of computers, number of cars, own bedroom, holidays abroad, dishwasher, and bathroom. Figure 1 shows item response category characteristics curves for each of the items. The probability of responding to category *k* is viewed as a function of family affluence, with affluence scaled in a standard normal metric. In this initial run, some categories were collapsed to achieve a stable estimate within each sample. For “bathroom”—those reporting 0 or 1 were collapsed, and those reporting 2 or 3 bathrooms were collapsed. Similarly for “computer”, having 0 or 1 computer was collapsed. It can be seen from Fig. 1 that at low levels of affluence having 0 or 1 computer is the most probable response. At higher affluence, responding 3 or more computers becomes the most probable response, increasing monotonously from medium to high, but reaching an asymptote at family affluence higher than a *Z-*score of 2 (Fig. 1).

**Fig. 2:**
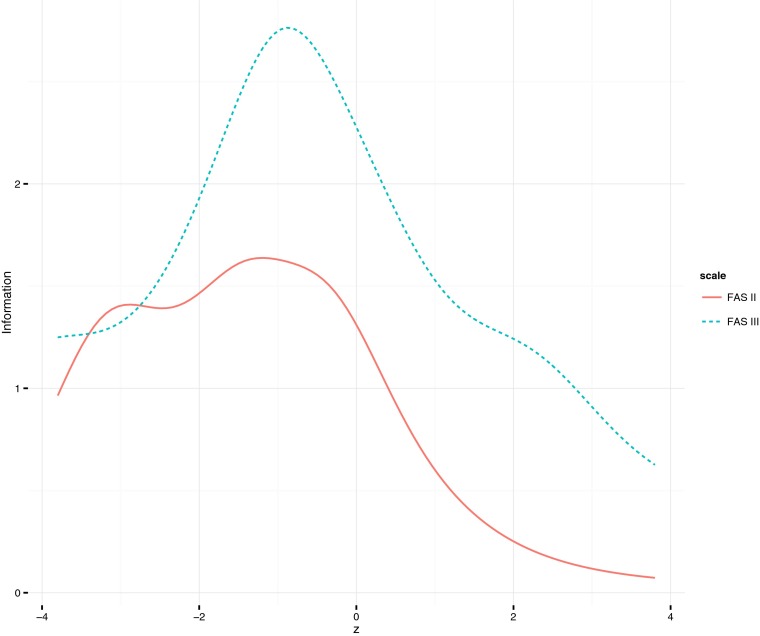
Test information curve for FAS II and FAS III

In the test of differential item functioning, estimation of item parameters for the dishwasher item did not converge in the Norwegian sample, likely due to few adolescents reporting not having a dishwasher. Thus for the for the initial DIF analysis of the complete six-item scale, the Norwegian sample was excluded. The DIF analysis of the six-item scale identified three anchor items, items that were invariant according to the DIF criterion: Computers, holidays abroad and dishwasher. To include the full sample, the analysis was repeated without the dishwasher item. The re-analysis again identified computers and holidays abroad as invariant items across all countries.

### Test Information and Reliability

To test whether the tested scale provided information across levels of affluence we computed test information curves (Lord 1980) as a function of family affluence, based on item parameter estimates obtained from the graded response model (Samejima 1997). To benchmark the revision, item information curves were also computed for the four-item FAS II scale. The test information curves are displayed in Fig. 2. It can be seen that the revised FAS III has peak information about the mean level of affluence. Importantly, the revised version captures the same amount of information on low levels of affluence, but clearly more information than FAS II at middle and high levels of affluence. At higher level of affluence the level of test information drops markedly.

Test-retest reliability for the FAS III was computed in the six countries that conducted a retest survey. The pooled test-retest correlation was *r* = 0.90. The test-retest reliability by country was 0.76 for Greenland, 0.81 for Norway, 0.87 for Italy, 0.89 for Scotland, 0.91 for Poland and 0.91 for Slovakia.

### Construct Validity

A valid measure of family affluence should relate to household income. As a crude test of criterion validity, FAS III summed scores were regressed on parent-reported income groups, stratified by country. The categorization of income groups differed across countries, and therefore had to be analysed separately. FAS scores correlated with parent reported income (Table 3), with Eta-squared close to 0.30 in most countries, but with weaker associations in Slovakia and Romania. In six of the eight countries the FAS III was more strongly related to income group than was the FAS II.

**Table 3:** Explained variance from FAS regressed on parent income group

Group	Eta^2^-FAS III	Eta^2^- FAS II
Denmark	0.32	0.24
Greenland	0.30	0.14
Italy	0.13	0.13
Norway	0.28	0.15
Poland	0.22	0.19
Romania	0.04	0.04
Scotland	0.31	0.27
Slovakia	0.09	0.05

## Discussion

In line with the objectives of the present study, the current results helped to identify a set of items that can be used to measure family wealth in international comparative surveys of adolescents. The results support a conservative revision of the FAS II, by keeping three of the original items from FAS II (Cars, Computer, Own bedroom), refining one of the items (Holidays abroad) and adding two new items (Dishwasher and Bathroom).

Consistent with the strategy in previous comparative studies of similar concepts (e.g. (Traynor and Raykov 2013)), the current study used a reflective measurement model to guide the revision of the family affluence scale. In each country, the bifactor analysis revealed a strong general factor, but also some specific factors. The observed general factor in the bifactor analysis is consistent with the notion that young people’s assets reflect an underlying dimension of family affluence. In comparison with the original FAS II scale the revised version, FAS III, provided more information on the high level of trait, and showed a stronger association with parental income. The shift towards information at a higher level of affluence was accomplished through the inclusion of the “bathroom” and “dishwasher” items. In studies using the consensual approach with similar indicators in 1999 (McKay 2004) only a minority regarded having a car (38 %), holiday abroad (19 %), home computer (11 %) and dishwasher (7 %) as necessities for life. It is interesting to note that none of these four indicators were judged as necessities in studies conducted in the UK in the late 90’s. To increase the face validity of the current study it would be valuable to know whether children and adolescents today see the included indicators as necessities, as such information could help to identify a cut-off point on the continuous affluence scale denoting a poverty line.

The test of differential item functioning suggested that at least three of the tested items were directly comparable indicators of affluence across countries. Importantly, two or more invariant items are the minimum requirement for construction of a comparable scale. Thus, these results provide some support for FAS III scale. The DIF analysis of the trimmed model identified national differences for three of the six indicators. Given the cultural, economic and geographical differences in the sampled countries, the observed differential item functioning was not surprising.

The test information functions indicated that the revised version of the scale (FAS III) provides more information on middle and higher end of the affluence trait than did the FAS II. In comparison with the FAS II scale, the information in medium and higher levels of affluence is an important step forward. In further development of the family affluence scale, items tapping information on the higher end of the distribution should be developed. The need for indicators higher affluence is especially relevant in countries where the target population is affluent. Notably, in Norway, ceiling effects were present for a number of items, indicating that these are too commonplace in a Norwegian context, as the level of wealth in Norway is very high. The pattern observed in the Norwegians sample highlights the need for future research to develop indicators that capture information about, and therefore have the potential to stratify the population at, the higher end of the affluence continuum. For research that focuses on the whole gradient of inequality from low to high affluence, or living standards in general, it is essential to capture an equivalent amount of information across the whole dimension of affluence.

A test of criterion validity revealed expected positive associations with parental income groups. The prediction was much weaker in the Slovak and Romanian sample. The weaker association in these samples might be related to methodological factors. The Romanian adolescent sample was smaller than the other samples, and the six income groups in the Romanian sample were relatively small, making estimates from this sample more prone to random fluctuation. In addition to these methodological differences, there are also other factors contributing to the observed variation. Household income is not the only source of affluence in a family, and there are cross-national differences in economic purchasing power, public support, and tax systems. For these reasons, a perfect association between household income and family affluence were not expected, and we would not expect completely uniform association across different countries. We do not have data to provide a specific explanation for the weak association in the Romanian and Slovak sample. Further studies of this association may reveal if these patterns can be generalized to the Romanian and Slovak population as a whole, and the reasons for this finding.

Schools selected for the current study were not sampled using a defined sample frame, and therefore the adolescents, by statistical definition, may not have been representative of the youth population in the participating countries. As the main research question was about the relationship between indicators and a theoretical concept, and not about the population characteristics per se (e.g. level of affluence in country A and B), the representativeness of the sample is unlikely to affect the results at this stage of scale revision. To extend the validity claims of the FAS III, however, further examination of the FAS III is recommended among nationally representative samples.

The current indicators were tested in European countries, and this limits the generalizability of the current results. Although countries outside Europe, such as the U.S. and Canada share features with the current sample, there might be differences in car ownership, and holidays abroad. To obtain the objective of a comparable scale, future research should therefore replicate the current findings in countries outside Europe.

In the current study, the final selection of items was obtained using criteria from psychometric measurement theory. Within social policy it has been shown that adopting a consensual approach to the definition of poverty, wealth or living standards can augment validity and interpretation of assets-based scales. An interesting avenue for future research would be to combine consensual and psychometric principles of item selection, and to use children’s and adolescents’ own views of necessities and desirable goods as criteria for item selection and scoring (Main and Bradshaw 2012). In particular a consensual approach would help to identify new indicators that provide information about high affluence. To obtain a better coverage of higher affluence one would need to expand the focus from not only necessities but also desirable goods, that a majority of adolescents would like to have, but do not have to have, to achieve an acceptable living standard.

The current study was motivated by the needs of a large-scale comparative international survey, and the demand for a brief six-item survey instrument. In studies with fewer constraints on the number of items, and with less emphasis on cross-national comparability, inclusion of country-specific indicators could optimize the measurement of family affluence further. The bifactor analysis revealed that a subset of the indicators work well in some countries, but poorly in other countries. For example, outdoor space was a relevant indicator in Denmark, Italy and Norway, but not in the other countries. These observed differences, call for developing country-specific indicators with higher local relevance.

## Conclusion

The current results indicate that a revised family affluence scale with comparable metric and better validity can be implemented in comparative studies. While originally developed in the context of health inequality research, the family affluence scale can be a valuable tool in many areas of research with children and young people when family affluence is a topic of relevance, also beyond the health field.

The FAS III provides an indirect way of assessing affluence among young people, and can be seen as a particularly relevant approach when parental income is difficult or impossible to obtain. Still, the current indicators should be regarded as a supplement to conventional indicators of SES, as the information covered by the scale belongs to a different domain than the information covered by conventional indicators of occupation or education (e.g. (Pförtner et al. 2015)).

## Appendix Main Variables Tested in the Current Study

**Table:**

Item variable name (history), item wording
Computer (FAS II) How many computers (PCs, Macs or laptops) does your family own?
Car, (FASII) Does your family own a car, van or truck?
(Holidays, FAS II) During the past 12 months, how many times did you travel away on holiday with your family?
(Own bedroom, FASII) Do you have your own bedroom for yourself?
(Holiday abroad, refined) How many times did you travel abroad for holiday/vacation last year?
(Outdoor space, new) Does your home have an outdoor space attached, (e.g. garden)?
(Own bed, refined) Do you have a bed of your own?
(Holidayhome, new) Does your family have a holiday (vacation) house/apartment?
(Dishwasher, new) Dishwasher
(Washer, new) Washing machine
(Dryer, new) Tumble dryer
(Internet, new) Do you have Internet access at home?
(Payed work, new) Do your parents pay people to do work in your home (e.g. cleaning, cooking, gardening)?
(Own computer, refined) Do you have your own computer?
(Bathroom, new) How many bathrooms (room with a bath) are in your home?
(IPod, new) Do you have an iPod or other personal music player?
